# In Silico Conformation of the Drug Colchicine into Tubulin Models and Acute Phytotoxic Activity on *Cucumis sativus* Radicles

**DOI:** 10.3390/plants11141805

**Published:** 2022-07-08

**Authors:** Omar Aristeo Peña-Morán, Jesús Jiménez-Pérez, Litzia Cerón-Romero, Maribel Rodríguez-Aguilar

**Affiliations:** 1 División de Ciencias de la Salud, Universidad Autónoma del Estado de Quintana Roo, Chetumal 77039, Quintana Roo, Mexico; maribel.rodriguez@uqroo.edu.mx; 2 División Académica de Ciencias Básicas, Universidad Juárez Autónoma de Tabasco, Cunduacán 86690, Tabasco, Mexico; jesus.jimpe1996@gmail.com (J.J.-P.); litzia.ceron@ujat.mx (L.C.-R.)

**Keywords:** colchicine, *Cucumis sativus*, microtubules, phytotoxicity, tubulin-models

## Abstract

Many tests are used to determine the toxic activity of miscellaneous substances, and those that are simple, fast, and inexpensive are useful for screening compounds with applications in different fields. The *Cucumis sativus* root growth inhibition test is an example of acute toxicity determinations. On the other hand, colchicine has been used as a herbicide to generate polyploids in plant species finally reaching the environment; for this reason, colchicine could become a point of attention in ecotoxicology. This work established that *Cucumis sativus*, at the colchicine binding site (CBS) in tubulin, shares 100% similarity with humans. Colchicine was docked on seven *Cucumis sativus* computational models of the αβ-tubulin heterodimer, allowing us to understand a possible conformation in tubulin to trigger its antimitotic effect. Furthermore, an in vitro phytotoxicity assay of colchicine-treated cucumber radicles indicated a hormetic-type concentration-dependent response with macroscopic changes in radicles and hypocotyl. These results support the highly preserved grade of tubulins in several species, and using microtubule inhibitors could require attention in ecotoxicological issues. The *Cucumis sativus* root growth test could help evaluate small molecules (colchicine analogs), chiefly by CBS interactions, a known druggable site, still a target in the search for antimitotic compounds.

## 1. Introduction

In the ecotoxicology field, many pharmaceutical products pollute the environment once used and excreted, both for humans and animals. For decades, observations in nature and laboratory investigations have provided evidence of drugs in water, sediments, sludge, etc. [1], which leads to exposure of living organisms in the environment to pharmaceutical products. However, much remains unknown about the effects of drugs on organisms in the ecosystem, particularly plants, and even more on the amounts of substances required to initiate a toxicological impact. Drug receptors in humans are, in most cases, highly conserved proteins between species, which may imply a similar mode of action, and published studies have used molecular docking as a potential tool to predict drug effects in aquatic organisms with exciting results [2].

Plant bioassays have become a valuable and efficient tool for monitoring toxicity in laboratory investigations due to their sensitivity, simplicity, and quick results [3]. In addition, these assays are characterized by the low cost of preparation and maintenance and the possibility of analyzing germination and growth parameters, such as germination rate, root elongation, biomass, or enzymatic activity [4,5]. Tests that positively or negatively evaluate radicle growth and seed germination have been used to assess physical, chemical, and biological agents [6] to study concentration-response relationships under specific and controlled conditions [7,8,9,10]. For this purpose, multiple plant species have been used to determine the phytotoxicity of physical, chemical, and biological agents, such as *Cucumis sativus* (cucumber) [11,12,13,14], *Lactuca sativa* (lettuce) [15], *Raphanus sativus* (radish) [16], *Trifolium pratense* (red clover) [17], *Medicago sativa* (alfalfa) [18], *Triticum aestivum* (wheat) [17,19], etc.

The root growth inhibition test has been used to explore the toxicity of compounds and extracts, and it has been used as an additional test to those carried out with the classic toxicity methods since the results have demonstrated a correlation with other models. An example of this was the 83% efficiency that the seed germination bioassay showed by detecting 5 out of 6 antitumor compounds [6] and a 100% efficiency when associated with the *Artemia salina* toxicity test with positive efficiency criteria when the mean Inhibitory Concentration (IC_50_) is less than 10 μg/mL [5,20].

The root growth inhibition test is a set of metabolic and morphogenetic processes in which plants pass through different phases until a photosynthetically active plant is formed. It lasts from metabolic reactivation to seedling development (radicle, hypocotyl, and cotyledons). Plant cells are divided by mitosis or meiosis, depending on the cell line type (somatic or germline). Both processes are relatively similar in animal and plant cells [21]. In the cell division process, chromatin condensation begins during the prophase, and chromosomes become visible and are aligned precisely on the metaphase plate in the middle of the mitotic spindle, which resembles a barrel shape because it lacks centrioles that are not necessary for cell division [22], and its fibers are fascicles of microtubules. In plants, cell division is determined by the phragmoplast, which consists of microtubules formed during late anaphase or early telophase [23,24].

Tubulins are globular proteins that form the mitotic spindle; they are created by heterodimers of α- and β-tubulins. Both tubulin sequences are very similar, with approximately 40% identity [25,26], and both are the main components of microtubules. Eukaryotic cell microtubules are among the three main cytoskeleton components and proteins with high sequence conservation in eukaryotic organisms [27]. They are highly dynamic structures that continuously alternate between their assembly (straight active structure) and disassembly (inactivate structure in curved form) [28]. The coexistence of growing and shrinking microtubules under the same conditions is termed “dynamic instability,” and their regulation is well understood [29]. The αβ-tubulin heterodimers target many molecules known as microtubule interfering agents (MIA); some are used in clinics for cancer treatment, and others in current investigations [30,31]. Furthermore, three different binding sites of microtubules are known: the colchicine binding site (CBS) (microtubule destabilizers), the vinca alkaloid binding site (microtubule destabilizers), and the taxane binding site (microtubule stabilizers) [32]. Currently, research on ligands binding the CBS on tubulin is ongoing.

**Figure 1:**
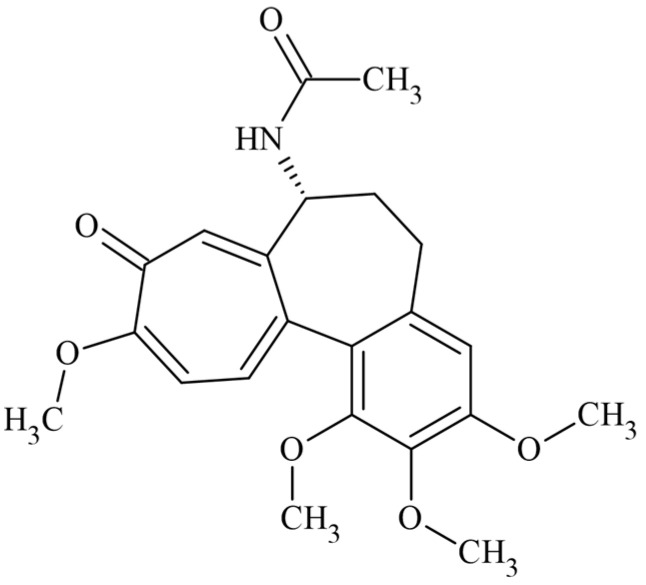
Chemical structure of the drug colchicine.

Colchicine (COL) is a naturally occurring active alkaloid from *Colchicum autumnale* (Figure 1). In the 1940s, its powerful antitumor effect was described; however, it was withdrawn from clinical studies due to its high cytotoxic activity [33]. It is currently used to treat gout and other inflammatory diseases [34,35,36]. COL is also a herbicide; it binds to tubulin dimers and inhibits microtubule formation. Loss of spindle microtubules affects nuclear division and chromosome separation. The lack of cortical microtubules interrupted cell and tissue morphogenesis [37]. COL binds reversibly and selectively to microtubules, particularly in the polar union of α- and β-tubulin, disturbing the mitotic spindle and causing microtubule depolymerization in eukaryotic cells. The compound has also been used for research in cytogenetics as an antimitotic due to its inhibitory effect on cell division in plants and animals, either allowing chromosomes to be observed or exploring several cell replications processes at the metaphase or anaphase [38].

*Cucumis sativus* is an annual herbaceous plant with a robust root system; seeds are approximately 0.8–1.0 cm long and 0.3–0.5 cm wide. The germination time is three to four days after sowing [39]. Cucumber seeds are easy to obtain due to their agricultural importance, have been previously used to evaluate the toxicity of several substances, and have been established as a COL-sensitive species [11,12,13,14,40]. However, the molecular toxicology that triggers the antimitotic effect of COL in the radicles of *C. sativus* has not been explained.

This work aimed to explain the binding mechanism of colchicine on *Cucumis sativus* radicles and models by in vitro and in silico approaches. The importance of this study lies in explaining the mechanism of action of COL in computational models of the α- and β-tubulin heterodimer of *Cucumis sativus*, informing how similar the dimer is compared with its homologues in humans since this could represent a point of attention for the environmental exposure of the drug COL and other inhibitors of tubulins in the CBS, as well as the potential ecological risk when COL is used as a herbicide. Furthermore, we used the root growth test as a low-cost, straightforward strategy for plant extracts and pure compound evaluations to detect those with cytotoxic potential, and this test could evaluate other types of molecules in different fields, including the pharmaceutical field.

## 2. Results and Discussion

### 2.1. Protein Sequence Alignment Comparison and Modeling of the αβ-Tubulin Heterodimers of C. sativus

Five α-tubulin chain sequences from cucumber aligned showed a high identity, and the average identity percentage (I%) was 90.2% ± 3.6%. Seven β-tubulin sequences from cucumber-aligned chains indicated an I% of 92.3% ± 1.5%. In both comparisons, a high I% was shared by the UniProt database sequences (the alignments can be observed in detail in Supplementary Materials in Tables S1 and S2).

The comparisons between the α- and β-tubulin chains from cucumber against human chains also showed high I% and similarity percentage (S%) (percentages and standard deviation can be observed in Table 1).

A high degree of evolutionary conservation of chains was found, which may be due to the animal and vegetable tubulins derived from a common ancestor [24,27]. Those results suggest a high degree of conservation between the primary protein sequences of both cucumber and human proteins.

The sequence of α-tubulin A0A0A0K6A8 showed the highest I% (84.7%, Table 1) and was applied for the α-tubulin chain modeling. All subsequent models of αβ-tubulin heterodimers were built with the A0A0A0K6A8 sequence because with the COL-interacting residues at the CBS in the human sequence (αS178, αT179, αA180, and αV181), only the αA180 residue was changed by the physiochemically similar residue αS180 for all five cucumber sequences.

The alignment of sequences at the CBS (only for the β-tubulin chain) was performed with the residues interacting with colchicine at a 4 Å distance. All seven β-tubulin cucumber sequences were aligned with the human β-tubulin sequence (UniProt: Q9BVA1). The I% (70.6%) and S% (100%) were obtained for all comparisons. The alignments of residues in the CBS can be observed in Table 2.

The TASSER models of tubulin macromolecules and the validation method results allowed us to know the overall quality of the models (the one α-tubulin model and the seven β-tubulin model) through Z-score calculations. The typical Z-score value for proteins around 445 residues lies between −3.9 and −13.0. Table 2 shows the Z-score for each β-tubulin model within the typical Z-score interval. The Z-score graphs of all models are detailed in Table S3, available in the Supplementary Materials. On the other hand, the Ramachandran plots of all models showed residues percentages in the favored and allowed regions of 96.2–97.5% limits. In addition, it was manually verified that none of the residues forming the CBS were in non-allowed regions. The Ramachandran plots for each model are available as Supplementary Materials (Table S3).

Comparative analysis of the β-tubulin chains between the human (Q9BVA1) and cucumber sequences revealed five changed residues: A250, M259, V315, A316, and I318 to S248, L257, A313, S314, and M316, respectively. The β-tubulin sequence fragment corresponding to the A0A0A0KQW7 UniProt entry replaced M318 (found in the rest of the sequences) with L316 (Table 2). For all seven *C. sativus* compared sequences, four sites with conservative (:) and one with semi-conservative (.) replacements were found.

The comparative results suggest that the CBS in all analyzed cucumber sequences was preserved compared to human sequences, particularly in the gene TUBB2B, which can be detected in many human tissues, mainly in the brain and male tissue [41]. These results could clarify why COL is used as a phytotoxic compound on many plant species. Proteins in the tubulin family have a high preservation degree, and minimal changes in the tubulin sequence have probably been imposed by the structural limitations of the assembly and disassembly of microtubules and the constraints imposed by the tubulin family association proteins such as kinesins and dyneins [27].

The crystallized structure of tubulin was obtained from the PDB database (4O2B, X-ray diffraction method, resolution: 2.3 Å). The crystallized model includes two heterodimers of α- and β-tubulin, a stathmin molecule, and tubulin-tyrosine ligase chains. In addition, because COL was co-crystallized, it was possible to situate the CBS and compare its conformation [42,43].

**Figure 2:**
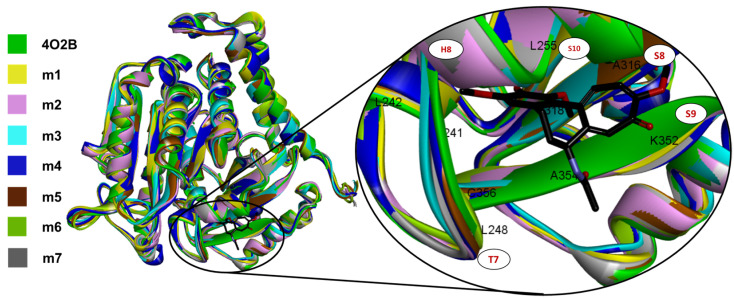
All seven TASSER αβ-tubulin models of *C. sativus* (each in different colors) aligned to 4O2B macromolecule, and a close-up view of the CBS with co-crystallized COL_4O2B_ (black sticks). Note that all models fit the tertiary structure of the 4O2B model, and qualitatively, the CBS is highly conserved.

The TASSER models with the highest C-score were selected, typically in (−5, 2), where a higher C-score value means a model with increased confidence and vice-versa [44,45,46]. An image of the seven aligned monomers of β-tubulin and a close-up view at the CBS showing the conformation adopted by the COL can be observed in Figure 2.

The CBS (named RB3-SLD by Ravelli et al., in 2004 [28]) is mainly found in the intermediate domain of the β-tubulin, placed between the S8 and S9 strands, the T7 turn, and the helices H7 and H8 (Figure 2). COL also interacts with the T5 loop of the α-tubulin chain mainly by αT179 electrostatic interactions, and this interaction stabilizes the αβ-tubulin heterodimer preventing tubulin from ongoing from a curved to a straight conformation.

The results of the sequence comparison between *Cucumis sativus* and *Homo sapiens* tubulins have suggested that proteins are presenting conserved regions, but also changed residues have been found in the CBS; however, these residues are semi-conservative or conservative, which could lead COL to obtain a stable conformation at the same mode already reported. In addition, the modeling and alignment originated seven heterodimers of αβ-tubulin from the protein sequences of *C. sativus*. Finally, an in silico molecular docking analysis was performed to understand how the COL could interact with the CBS.

### 2.2. In Silico Docking Studies between Colchicine and the C. sativus αβ-Tubulin Models

**Figure 3:**
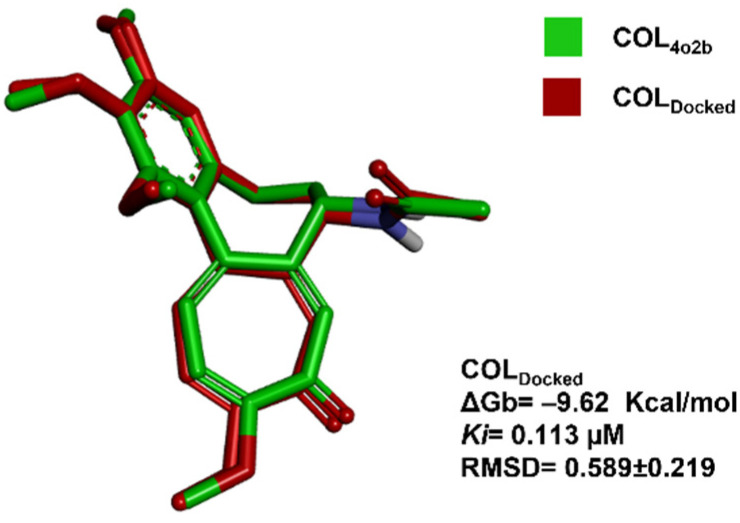
Molecular docking validation. Image of ligand poses between the co-crystallized ligand (COL_4O2B_, green) [47] and the mean-energy pose found with the docking algorithm (COL_Docked_, red) are shown.

The in silico molecular docking validation analysis with COL (COL_Docked_) showed a cluster of lowest energy of 53/7 (poses in the lowest energy cluster/total number of clusters). The pose with the lowest energy at the cluster, the mean energy in the cluster, *Ki*, and RMSD computed by comparison with co-crystallized COL_4O2B_, are shown in Figure 3.

The atomic details of the CBS were revealed in 2004 when Ravelli et al., published the structure of tubulin with the stathmin-type domain of the RB3 protein in the colchicine complex (PDB: 1SA0); however, higher resolution structures were obtained with tubulin–tyrosine ligase [47]. Superimposition between the colchicine conformation previously reported by Prota et al. in 2014 and the conformation obtained by docking analysis with the *C. sativus* model was performed. It can be visualized in Figure 3. The lowest mean energy conformer of COL_Docked_ was found like that already reported in the PDB as 4O2B crystal by Prota et al. in 2014. Once the RMSD at the validation analysis was <2 Å, the docking parameters were acceptable for further investigations.

The molecular docking analysis of the CBS with the COL ligand and all seven αβ-tubulin models from *C. sativus* showed 8 to 14 conformational clusters after 100 runs. For those with the lowest conformational energy, the mean free Gibbs energy (ΔGb in Kcal/mol), the inhibition constant (*Ki*) obtained from the standard Gibbs free energy equation, and the RMSD computed for each pose in the cluster by the COL_4O2B_ comparison are reported in Table 3. For the models m5, m6, and m7, a low number of poses was obtained in the lowest energy cluster. The rest of the models obtained between 26–41 poses. The energy interval obtained for COL in all *C. sativus* models was about −8.88 ± 0.58 Kcal/mol with a percentage coefficient of variation of 6.54%.

Differences in primary sequence can be found in each model of the β-tubulin proteins from *C. sativus* (Table S2). However, the COL affinity of binding into the CBS is not affected. In addition, the in silico model minimization did not affect the pocket of binding. Furthermore, all conformations in the lowest energy cluster are like that adopted by COL in the 4O2B crystal (RMSD < 2 Å). As previously reported, those results suggest that COL could bind to CBS in *C. sativus* tubulin and consequently giving a phytotoxicity induction effect and antimitotic activity [11,12,13,14,40].

**Figure 4:**
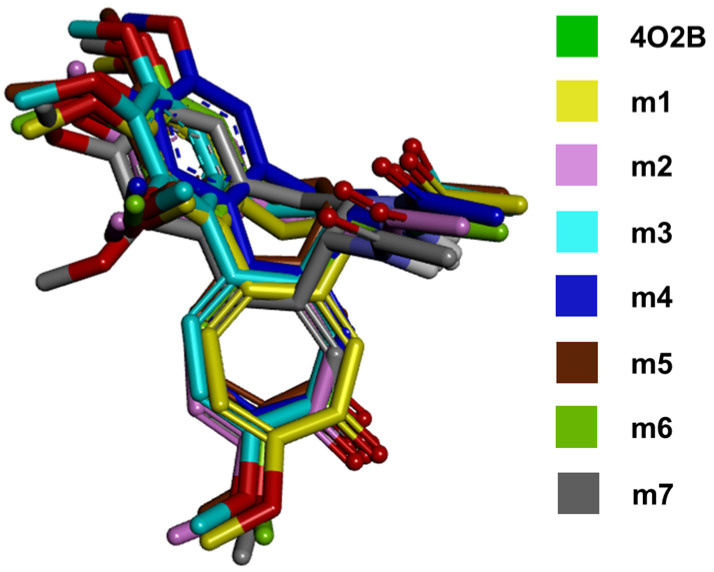
All COL poses merged. The figure shows the lowest-energy conformations achieved from molecular docking of COL with the αβ-tubulin models from *C. sativus*. The conformations showed that the orientation of the functional groups is maintained in the CBS compared with the control (4O2B).

Figure 4 compares the lowest energy conformation adopted by the COL of all the models used in this study. The complete interaction network between COL and the models m1–m7 can be observed in Table S4. In addition, the 4 Å interacting residues of αβ-tubulin are shown in Table S4.

The results in the in silico molecular docking between COL and CBS in the cucumber model suggest a strongly related conformation in the 4O2B model. Furthermore, the alignment between COL conformations obtained in this work (Figure 4) and that found in the crystallized structure 4O2B also suggests that changes in the residues between cucumber and human chains do not affect the binding affinity due to the conserved physicochemical environment of amino acids in *C. sativus* sequences (conservative and semi-conservative). All complete interaction maps can be seen in Table S4 in the Supplementary Materials. Finally, the root growth inhibition test was performed with the germinated radicles of *C. sativus* to explore an in vitro concentration-dependent activity by COL.

### 2.3. Technical Considerations before Starting the Phytotoxic Test

**Figure 5:**
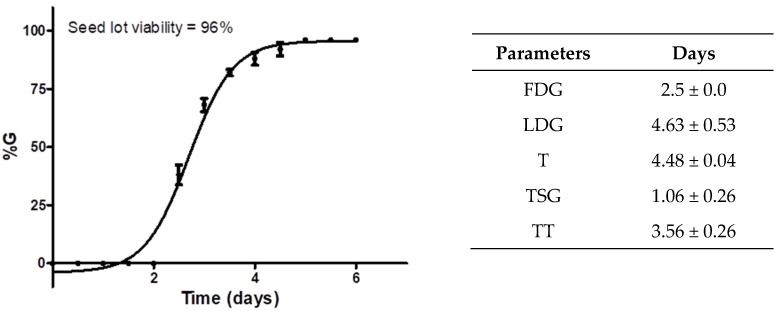
Cucumber seed germination parameters were calculated according to a previous phytotoxicity assay. The graph shows the germination percentage (%G) of the seed lot of *C. sativus* against germination days, lot viability, and calculated germination factors. Each point in the graph represents the %G ± standard deviation (*n* = 50) against time (days).

The viability of cucumber seeds was calculated to be 96% (*n* = 50), showing a high seed viability rate in the lot. Additionally, the treatment time (TT) for the COL treatment was 3.56 days (85 h) after sowing. All assessed parameters can be observed in Figure 5. A high germination percentage was crucial for the phytotoxicity test because it is based on root elongation and not germination capacity.

The first day of germination (FDG) indicates how fast the germination begins. For the sown seeds of *C. sativus,* this time was determined in 2.5 days for the detection of the first germinated seeds; for the LDG (last day of germination), the result indicated germination of 96% of the seeds at 4.63 h, which showed a difference of 2.1 days between the beginning and the end of germination; the average germination time (T) indicated the time required for viable seeds to germinate, which was found to be approximately 4.5 days (about 108 h); the mean seed germination time (TSG) and FDG allowed us to detect the best treatment time (TT) to start the trial with COL.

### 2.4. In Vitro Antimitotic Effect of Colchicine (Phytotoxicity)

**Figure 6:**
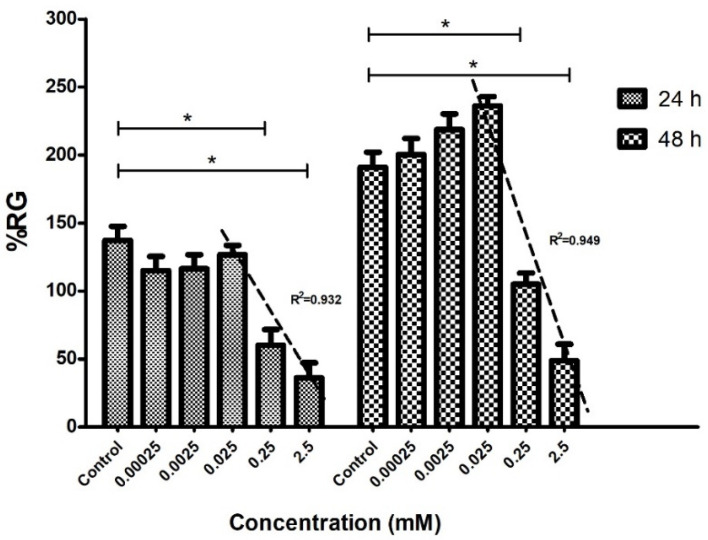
In vitro antimitotic effect of colchicine on cucumber radicles. The graph indicates the average percentage of primary root growth (%RG) for each concentration of COL on the *Cucumis sativus* radicles (*n* = 20) at 24 and 48 h of incubation. Concentrations of 2.5 and 0.25 mM showed significant differences (* *p* < 0.05) compared to the control at 24 and 48 h of treatment. The three highest concentrations were applied for linear regression analysis. The IC_50_ was determined for each quantified day. The regression coefficients of determination (R^2^) in the graph were relatively close to the unit and can be observed for each analysis.

Phytotoxicity assessment has enabled the potential use of COL as an inhibiting radicle growth agent in cucumber seeds. The quantitative results in Figure 6 strongly suggest that cucumber radicle growth was affected by treatment with COL in a concentration-dependent approach; furthermore, the compound could induce hormesis after 48 h of treatment. Thus, it has been established that hormesis is related to stress in living things, the sum of non-specific biological responses to threatening stimuli and tends to disrupt homeostasis. Hormesis is currently considered a non-specific adaptive response to low doses of stressors [48].

At a concentration of 0.025 mM, a stimulating effect of radicle growth was observed; however, phytotoxic effects were observed at 0.25 mM after the first 24 h of incubation. The COL-determined mean growth inhibitory concentration (IC_50_) values at 24 h and 48 h of treatment were 0.85 mM and 1.77 mM, respectively. In addition, the results of Figure 6 showed that the minimum tolerable concentration in the *C. sativus* radicles was 0.025 mM of COL since this concentration does not significantly affect the elongation of the radicles.

Radicle growth in cucumber germinated seeds could be used to screen for in vitro antimitotic activity. A work limit concentration of 1 mg/mL (2.5 mM COL equivalence) is recommended. COL has been used in phytotoxic concentration intervals from 0.25 mM to 38 mM to induce somatic polyploidies in vitro [49].

Although a large arsenal of compounds targeting microtubules is known [50,51], new compounds targeting the CBS continue to be researched in depth due to the potential druggable sites in microtubules, representing a possibility for anticancer drug development [33]. However, the possible ecotoxicological impact that cytotoxic compounds could have on the environment, such as those affecting the microtubules of cells, should be considered.

### 2.5. Macroscopic Characteristics of Cucumber-Treated Seeds

**Figure 7:**
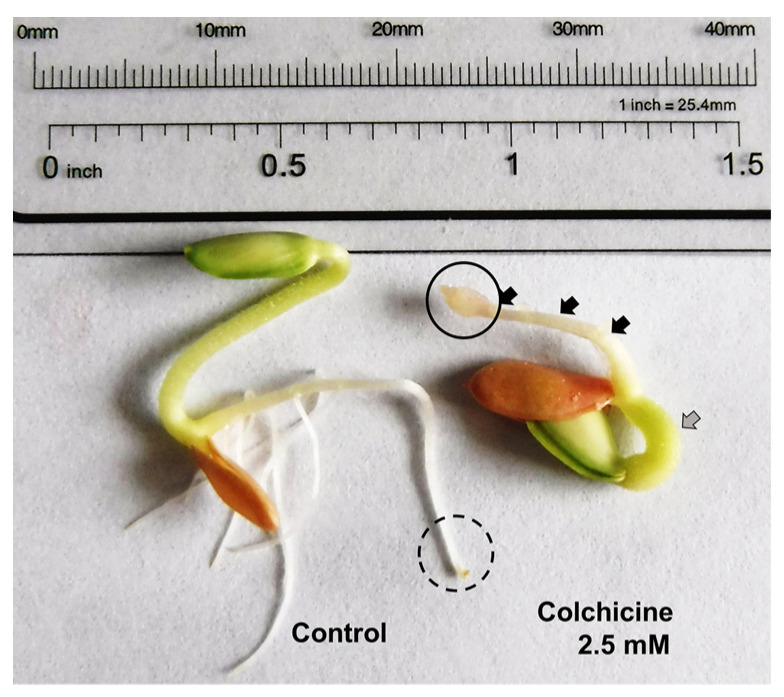
Macroscopic and microscopic analysis of control and treated germinated seeds of cucumber. The germinated control (seed on the left) shows the typical morphology of the primary and secondary roots, tegument, cotyledon, and hypocotyl can be observed. A morphological change can be observed in the germinated seeds treated with 2.5 mM of COL (seed on the right), a broader and shorter hypocotyl (gray arrow), and primary root blebbing formation of approximately 1–2 mm (solid circle), and secondary root inhibition (black arrows).

Macroscopic observations showed interesting results. The morphology of two representatives of germinated seeds was observed: control group (Figure 7, left) and post-treatment with 2.5 mM COL for 48 h (Figure 7, right). The COL treatment morphological change suggested an antimitotic effect by decreasing the growth of cucumber radicles.

A blebbing formation can be observed at the division zone of the primary root in COL-treated radicles (Figure 7, right), and it is associated with the phytotoxic effect described for COL. Dicotyledonous plants have a primary root that repeatedly branches to generate lateral roots that originate exclusively from pericycle founder cells. These are initiated when individuals or pairs of pericycle founder cells undergo several rounds of anticlinal and periclinal divisions, followed by patterning and emergence, activation of the new meristem, and lateral root elongation [52]. COL causes lateral root cell division inhibition and decreased radicle growth on germinated cucumber seeds, previously reported as polyploid formation in other plant species [53].

**Figure 8:**
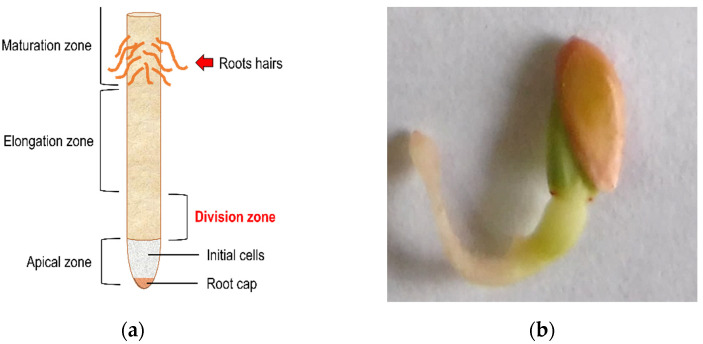
Blebbing formation in the division zone. (**a**) Indicates the segments and names assigned to a radicle [23]. (**b**) The figure suggests a *C. sativus* seed treated by COL 2.5 mM and the blebbing formation in the division zone.

What is sought with the root growth test is a statistically significant decrease in the size of the radicles of *C. sativus* and a concentration-dependent effect on the IC_50_ calculation; those parameters make it possible to find the phytotoxic activity of COL. Additional observations came from some morphological changes in the radicles by COL treatment. As it was described in Figure 8, a blebbing formation can be observed at the division zone of the primary root in COL-treated radicles, and it is associated with the antimitotic effect described for COL that causes the inhibition of the lateral root division and decreases radicle growth on germinated cucumber seeds, previously reported as polyploid formation in other plant species. [53] Figure 8 shows the components in a primary root (Figure 8a), and a *C. sativus* COL treated seed (Figure 8b). The comparison revealed that blebbing formation could be found at the division zone in radicles, consistent with the effects of an antimitotic compound when root growth is also decreased.

The conservation of the significant characteristics of cell organization and several genes in eukaryotic organisms strengthens the theory that all existing eukaryotic forms have evolved from a common ancestor [54]. For example, plant cells are derived from undifferentiated cells called meristematic cells, and through germination or seedling formation, they undergo constant cell division to become a complete plant.

Nowadays, it is unknown how COL treatments affect meristematic cells in roots. However, the mechanism of action has been widely studied; it was even more challenging to find crystallized and elucidated structures of the tubulin-COL complex from *C. sativus* in the Protein Data Bank repository. However, the TASSER models have made it possible to understand the conformation that COL could adopt between the α- and β-tubulin chains in the *C. sativus* tubulin models; even more, the minimal changes of residues found at the sequences were conservative for the COL docking. According to the results of this study, the COL mechanism of action between human and plant (cucumber) models were similar, which could represent an ecotoxicological risk depending on the concentration that can be found in the environment.

The authors consider that the root growth inhibition assay could be a speedy assay to explore compounds with antimitotic effects at the CBS in tubulin: applying the test compounds at 1 mg/mL on a group of germinated seeds, then analyzing the morphological changes after the treatment (at least 24 or 48 h) and comparing them against a control group. It is recommended to examine the viability of the seed lot and determine the FDG, LDG, %G, T, TSG, and TT, to perform treatments with the most significant number of germinated seeds. Advantages of this trial as a research strategy include low-cost, easy assembly, and straightforward interpretations.

## 3. Materials and Methods

### 3.1. Protein Sequence Alignment Comparison and Modeling of the αβ-Tubulin Heterodimer of C. sativus

A comparative bioinformatic analysis was performed with the α- and β-tubulin sequences between humans and cucumbers (*Homo sapiens* vs. *Cucumis sativus*). In the UniProt database, five protein sequences corresponding to α-tubulin (UniProt: A0A0A0K6A8, A0A0A0LFM5, A0A0A0KWR8, A0A0A0KWB5, and A0A0A0KIM4) and seven sequences corresponding to β-tubulin (UniProt: A0A0A0L2I9, A0A0A0LTS3, A0A0A0LCY8, A0A0A0LPG6, A0A0A0LXT7, A0A0A0LVT8, A0A0A0KQW7, and) were found, all sequences belonged to *C. sativus*. The α- and β-tubulin sequences from UniProt were aligned with those from humans: α-tubulin chain 1B (UniProt: P68363) and β-tubulin chain 2B (UniProt: Q9BVA1), respectively. The percentage of similarity (S%) and identity (I%) were obtained by the UniProt Basic Local Alignment Search Tool (BLAST) [55]. In situ, the residues forming the CBS [47] in both α- and β-tubulin were aligned for identity and similarity determinations. The S% was calculated considering the radical-conservative replacement ratios, volume, and polarity between residues [56]. Residues forming the CBS (4 Å) were as follows: αS178, αT179, αA180, αV181, βC241, βL248, βA250, βD251, βK254, βL255, βN258, βT314, βV315, βA316, βI318, βN350, and βK352.

One α-tubulin monomer of *C. sativus* was modeled using the α-tubulin sequence A0A0A0K6A8 (due to its highest identity compared to the human α-tubulin protein); likewise, all seven β-tubulin sequences from *C. sativus* found in the Uniprot database were modeled. The TASSER server was used for this purpose [44,45,46]. In addition, the PDB model of the α-tubulin chain from *Homo sapiens* (PDB: 5IJ0) [57] and the *Bos taurus* β-tubulin chain co-crystallized with COL [43] (PDB: 4O2B; the β-tubulin chain in the 4O2B model from the PDB database shares 445 identical residues (100% identity) with the human β-tubulin chain in the UniProt alignment of Q9BVA1 vs. Q6B856) were used as templates to guide the modeling.

The protein models of α- and β-tubulin chains of *C. sativus* were individually validated with the Z-score, indicating overall model quality. The ProSa-web service obtained a Z-score for each model [58,59]. In addition, Ramachandran plots were obtained with the Discovery Studio v3.5 software [60] to visualize energetically allowed regions for backbone dihedral angles ψ (psi) against φ (phi) of amino acid residues, and the favored and allowed amino acids were determined and reported as a percentage [61]. Once all monomers were validated in biomolecular in silico experiments, seven models of αβ-tubulin of *C. sativus* were obtained with the guanosine triphosphate (GTP) and COL ligands. All β-tubulin single models were merged into a heterodimer with α-tubulin using the matchmaker tool for comparison from the UCSF Chimera v1.6 software [62] and successively energy-minimized in the OPLS_2005 force field using the accessible version of Maestro v9.6 software [61].

### 3.2. In Silico Docking Studies between Colchicine and the C. sativus αβ-Tubulin Model

The COL model was optimized in an entire energy minimization force field MMFF94 (Avogadro software v1.1.1) [63] and docked into the CBS of the crystallized model 4O2B, and all seven validated αβ-tubulin models of *C. sativus* with Autodock v4.2 [64]. The polar hydrogen atoms were configured with Kollman (AMBER) charge assignment for the docking analysis preparation. A grid of 13.9 angstroms per side (37 points) was situated in the colchicine binding site in the tubulin heterodimer. One hundred runs of a Lamarckian genetic algorithm were configured. The Root Mean Square Deviation (RMSD) tolerance of 2.0 Å was used to create the conformational cluster. The poses in the cluster with the lowest energy (Gibbs free energy (ΔGb), and inhibition constant (*Ki*), were reported) were analyzed. The Discovery Studio v3.5 software [60] was used to visualize and analyze interactions in the poses network with the lowest binding energy at each model.

Additionally, the RMSD of each pose in the lowest energy cluster was obtained by aligning to the conformation adopted by the COL crystallized conformation described by Prota et al., 2014 [43] and published with PDB ID: 4O2B (COL_4O2B_).

### 3.3. Technical Considerations before Starting the Phytotoxic Assay

#### 3.3.1. Before Sowing

*Cucumis sativus* seeds were purchased from Germinal^®^ (germinal.com.mx). Before the test, an inspection was carried out, and using seeds and teguments with no damage was essential to ensure a high percentage of germination. Briefly, seeds were washed with neutral detergent and distilled water to remove polluting particles, additives maintaining embryo viability, hormones for seed dormancy, as well as fungal growth prevention.

For *C. sativus* seed germination, Petri dishes (SYM, 75 × 15 mm) and filter paper as a substrate were used, and the seeds (*n* = 25, in duplicate) were hydrated with purified water (0.1 mL/cm^2^) and incubated in a manufactured incubator at room temperature (26.3–31.4 °C) in darkness [65]. Cucumber radicles were counted and photo-documented every 12 h with a FinePix XP120 camera (FUJIFILM^®^, Villahermosa, Mexico).

#### 3.3.2. After Sowing

The first day of germination (FDG) and the last day of germination (LDG) were obtained; additionally, some germination factors were calculated [66,67,68]:

The germination percentage (%G) provides the viability of the seed lot (Equation (1)).

(1) $$\%G=\frac{n_{i}}{N}100$$

where n_i_ = number of seeds germinated at the time i and N = number of seeds sown.

The average germination time (T) measures the seeds’ average germination time to germinate (Equation (2)).

(2) $$T=\frac{\sum \left(n_{i}t_{i}\right)}{\sum n_{i}}$$

where n_i_ = number of seeds germinated at the time i, and t_i_ = number of days after sowing.

The half-time spread of germination (TSG) is the average time between the seed lot’s first and last germination event (Equation (3)).

(3) $$\mathrm{TSG}=\frac{\mathrm{LDG}\;-\;\mathrm{FDG}}{2}$$

where LDG is the time (days) for the first visible radicle germinated and FDG is the time (days) for the last observed radicle.

Treatment time (TT) is the germination time required to begin the phytotoxicity test (Equation (4)).

(4) TT=FDG+TSG

In this work, the TT parameter was used to assess the acute phytotoxic effect of COL on germinated seeds of *C. sativus*.

### 3.4. In Vitro Phytotoxic Effect of Colchicine

A stock was prepared with 1.05 mg/mL (2.5 mM) of COL (Sigma^®^ Cat. C9754, ≥95% purity HPLC powder). The volume to make serial dilutions (1:10) and the volume used in Petri dishes (0.1 mL/cm^2^) for the treatment must be considered. Five exponential dilutions (2.5 mM, 0.25 mM, 0.025 mM, 2.5 μM, and 0.25 μM) were tested to induce a concentration-dependent phytotoxic effect by COL. In addition, a growth control (purified water) was included in the assay.

Twenty-five cucumber seeds were sown in duplicate for each Petri dish (SYM, 75 × 15 mm). COL treatment was initiated following the TSG. The radicles were photo-documented before treatment initiation (zero hours) and subsequently at 24 and 48 h post-treatment. The images were analyzed with GIMP 2 v10.6 software [69], and the root area in pixels was measured for each radicle to obtain the average percentage of primary root growth (%RG) (Equation (5)).

(5) $$\%\mathrm{RG}=\frac{\sum A_{x}\;}{\sum A_{0}}\;\times \;100$$

where A_x_ is primary root area of treated seeds, A_0_ is primary root area of control seeds; measured radicles (N) are in the same count in both treated and control seeds in the formula.

The data obtained at each concentration evaluated were normalized, and the first ten data points, including defective radicles and non-germinated seeds, were removed. Next, the average and standard deviation of the twenty-intermediate data (2nd and 3rd quartiles) were calculated for the remaining forty measured radicles. Finally, the COL logarithmic concentrations and %RG were graphed. A one-way analysis of variance (ANOVA) with a Dunnett correction was performed, and a significant difference was considered when *p* < 0.05 in a 95% confidence interval. In addition, the mean inhibitory concentration (IC_50_) was assessed by interpolation (Y = 50%) in the straight-line equation at the semilogarithmic graph.

## 4. Conclusions

In conclusion, the results presented in the current study indicated that the phytotoxic effects of COL on cucumber root growth are dependent on the concentration, which could be explained by the high conservation degree of the mitotic spindle proteins between human and cucumber species (similarity = 100%). Furthermore, the CBS is situated between the interface of α- and β-tubulin monomers, which, when docked with COL, has enough affinity to form the colchicine-tubulin complex, promoting microtubule destabilization and, as a macroscopic effect, a decrease in the primary root length from cucumber seeds, a blebbing formation in the division zone of the radicles, as well as thickening of the hypocotyl and inhibition of secondary roots. Additionally, the average concentration that affects the morphology of the plant (0.85 mM and 1.77 mM at 24 and 48 h of exposure) was described, together with the minimum tolerable concentration to prevent the growth of the primary radicle (0.025 mM). Using COL as a herbicide could represent a soil risk due to its antimitotic action mechanism, affecting plants and terrestrial and aquatic organisms.

## Figures and Tables

**Table 1:** Alignment of complete α- and β-tubulin sequences of *C. sativus* against *H. sapiens* (P68363 for α-tubulin chain and Q9BVA1 for β-tubulin chain).

Entry	Length	Identity (%)	Similarity (%)
**α-tubulin**			
A0A0A0K6A8	450	84.7	97.6
A0A0A0LFM5	449	81.8	96.7
A0A0A0KWR8	449	81.4	96.7
A0A0A0KWB5	450	84.0	98.0
A0A0A0KIM4	415	77.8	97.6
Mean	442.6 ± 15.4	82.0 ± 2.7	97.3 ± 0.6
**β-Tubulin**			
A0A0A0L2I9	446	84.5	95.7
A0A0A0LTS3	445	83.6	96.4
A0A0A0LCY8	446	83.0	95.5
A0A0A0LPG6	449	83.3	96.4
A0A0A0LXT7	447	83.4	94.6
A0A0A0LVT8	443	86.1	96.6
A0A0A0KQW7	441	82.9	97.1
Mean	445.3 ± 2.6	83.8 ± 1.1	96.1 ± 0.8

**Table 2:** In situ protein sequence alignment between the residues forming the CBS, Z-score, and percentage of residues in the Ramachandran plot analysis within favored and allowed regions.

Uniprot ID	β-Tubulin Residues in the CBS																	Model	Z-Score	RP ^1^ (%)
	238	241	242	248	250	251	254	255	258	259	314	315	316	318	350	352	378			
**Q9BVA1**	V	C	L	L	A	D	K	L	N	M	T	V	A	I	N	K	I	-	-	-
**A0A0A0L2I9**	V	C	L	L	S	D	K	L	N	L	T	A	S	M	N	K	I	m1	−9.36	96.2
	*	*	*	*	:	*	*	*	*	:	*	.	:	:	*	*	*			
**A0A0A0LTS3**	V	C	L	L	S	D	K	L	N	L	T	A	S	M	N	K	I	m2	−10.01	96.4
	*	*	*	*	:	*	*	*	*	:	*	.	:	:	*	*	*			
**A0A0A0LCY8**	V	C	L	L	S	D	K	L	N	L	T	A	S	M	N	K	I	m3	−9.65	96.4
	*	*	*	*	:	*	*	*	*	:	*	.	:	:	*	*	*			
**A0A0A0LPG6**	V	C	L	L	S	D	K	L	N	L	T	A	S	M	N	K	I	m4	−9.95	96.9
	*	*	*	*	:	*	*	*	*	:	*	.	:	:	*	*	*			
**A0A0A0LXT7**	V	C	L	L	S	D	K	L	N	L	T	A	S	M	N	K	I	m5	−9.39	96.6
	*	*	*	*	:	*	*	*	*	:	*	.	:	:	*	*	*			
**A0A0A0LVT8**	V	C	L	L	S	D	K	L	N	L	T	A	S	M	N	K	I	m6	−9.48	96.6
	*	*	*	*	:	*	*	*	*	:	*	.	:	:	*	*	*			
**A0A0A0KQW7**	V	C	L	L	S	D	K	L	N	L	T	A	S	L	N	K	I	m7	−9.81	97.5
	*	*	*	*	:	*	*	*	*	:	*	.	:	:	*	*	*			

^1^ Ramachandran Plot. (*) Same residue. (:) Conservatives residues. (.) Semi-conservative residue.

**Table 3:** Results from the molecular docking analysis into the CBS between COL and the seven αβ-tubulin heterodimer models of *C. sativus*.

Model	Poses/Clusters ^1^	ΔGb (Kcal/mol)	Ki (µM)	RMSD
m1	28/8	−8.99 ± 0.59	0.412	1.12 ± 0.39
m2	26/10	−8.79 ± 0.45	0.606	1.11 ± 0.31
m3	30/9	−9.56 ± 0.51	0.145	1.01 ± 0.35
m4	41/8	−9.65 ± 0.21	0.091	1.18 ± 0.79
m5	2/10	−8.44 ± 1.01	1.195	1.02 ± 0.57
m6	2/14	−8.72 ± 1.02	0.744	1.01 ± 0.51
m7	4/10	−8.03 ± 0.67	1.988	1.58 ± 0.26

^1^ Poses in the lowest energy cluster/total number of clusters.

## Data Availability

Not applicable.

## Supplementary Materials

The following supporting information can be downloaded at: https://www.mdpi.com/article/10.3390/plants11141805/s1; Table S1: Identity percentages from the comparison of the α-tubulin sequences of *C. sativus* found in the UniProt database. Table S2: Identity percentages from the comparison of the β-tubulin sequences of *C. sativus* found in the UniProt database. Table S3: Alignment of α- and β-tubulin sequences of *C. sativus* against *H. sapiens* sequences (P68363 for α-tubulin chain and Q9BVA1 for β-tubulin chain). Table S4. Interaction diagrams between *C. sativus* tubulin models and the lowest-energy conformation of each cluster.
