# Supplementary material for: In Silico Conformation of the Drug Colchicine into Tubulin Models and Acute Phytotoxic Activity on Cucumis sativus Radicles
Source: Plants (Basel). 2022 Jul 8;11(14):1805. doi: 10.3390/plants11141805 (PMC9323635; doi:10.3390/plants11141805)
Supplement: Supplementary file 1 [file plants-11-01805-s001.zip › plants-1804801-supplementary.pdf]

In Silico Conformation of the Drug Colchicine into Tubulin Models and Acute Phytotoxic Activity on Cucumis sativus Radicles

Omar Aristeo Peña-Morán*, Jesús Jiménez-Pérez, Litzia Cerón-Romero, Maribel Rodríguez-Aguilar

Supplementary material

Table S1. Identity percentages from comparing α-tubulin sequences of *C. sativus* found in the UniProt database.

**Identity (%)**	**A0A0A0K6A8**	**A0A0A0LFM5**	**A0A0A0KWR8**	**A0A0A0KWB5**	**A0A0A0KIM4**
A0A0A0K6A8	100				
A0A0A0LFM5	90.4	100			
A0A0A0KWR8	92.0	94.9	100		
A0A0A0KWB5	96.0	90.4	91.3	100	
A0A0A0KIM4	89.5	84.8	85.3	88.0	100

Table S2. Identity percentages from the comparison of the β-tubulin sequences of C. sativus found in the UniProt database.

**Identity (%)**	**A0A0A0L2I9**	**A0A0A0LTS3**	**A0A0A0LCY8**	**A0A0A0LPG6**	**A0A0A0LXT7**	**A0A0A0LVT8**	**A0A0A0KQW7**
A0A0A0L2I9	100						
A0A0A0LTS3	92.15	100					
A0A0A0LCY8	**95.75**	91.93	100				
A0A0A0LPG6	92.89	92.65	92.20	100			
A0A0A0LXT7	93.06	92.84	92.17	91.33	100		
A0A0A0LVT8	94.39	93.93	92.82	93.10	93.51	100	
A0A0A0KQW7	90.60	90.78	90.81	88.64	90.60	91.42	100

Table S3. Ramachandran plot analysis for models of α- and β-tubulin proteins (left graphs). ProSA-web z-scores of all protein chains in PDB are determined by X-ray crystallography (light blue) or NMR spectroscopy (dark blue) concerning their length. The z-scores of calculated models of α- and β-tubulin proteins are highlighted as large dots (right graphs).

<table>
<tr><th>Chain
UniProt ID
Model</th><th>Ramachandran plot</th><th>z-scores</th></tr>
<tr><td>α-tubulin
A0A0A0K6A8
---</td><td>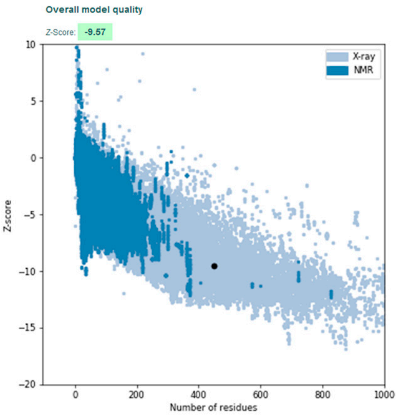
</td><td>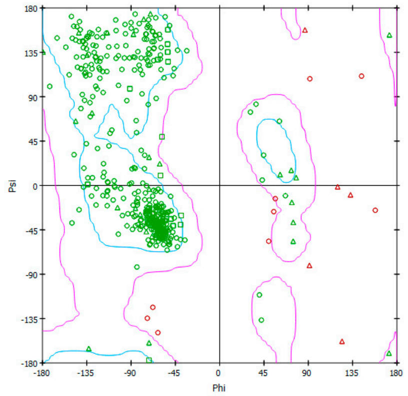
</td></tr>
<tr><td>β-tubulin
A0A0A0L2I9
m1</td><td>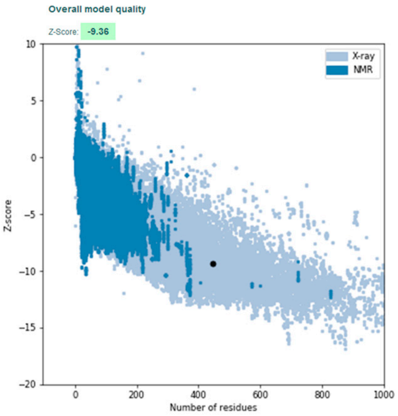
</td><td>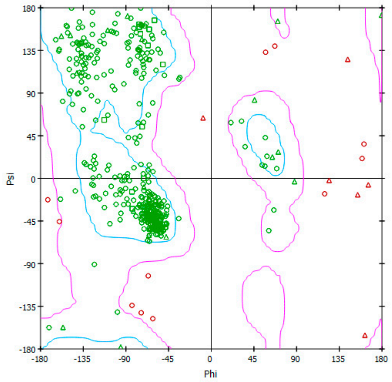
</td></tr>
<tr><td>β-tubulin
A0A0A0LTS3
m2</td><td>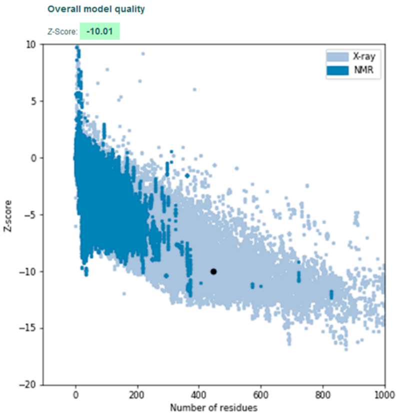
</td><td>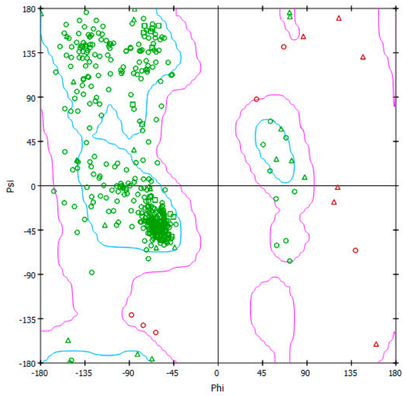
</td></tr>
</table>

β-tubulin A0A0A0LCY8 m3	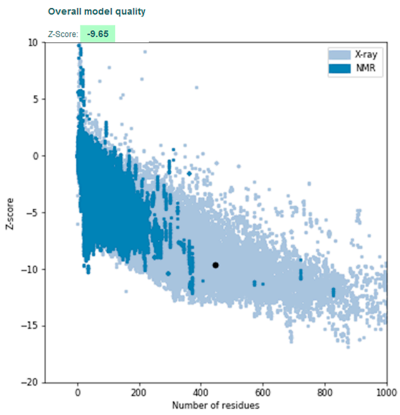	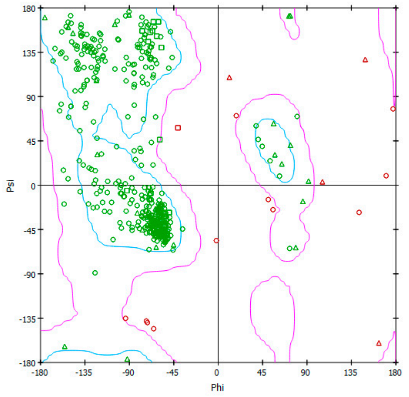
β-tubulin A0A0A0LPG6 m4	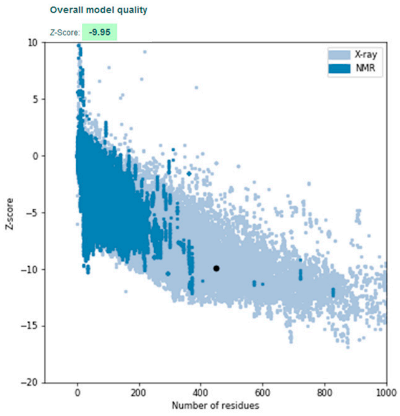	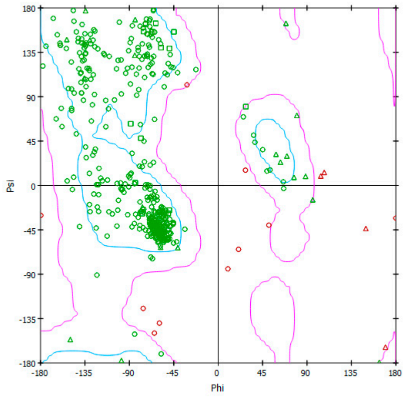
β-tubulin A0A0A0LXT7 m5	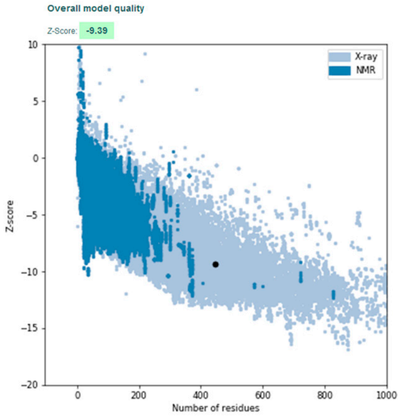	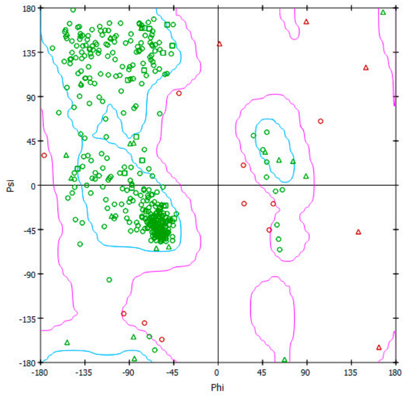

β-tubulin A0A0A0LVT8 m6	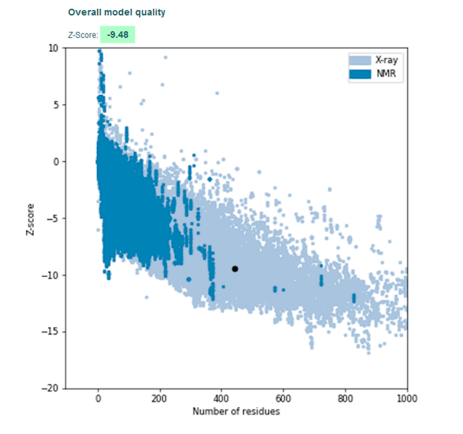	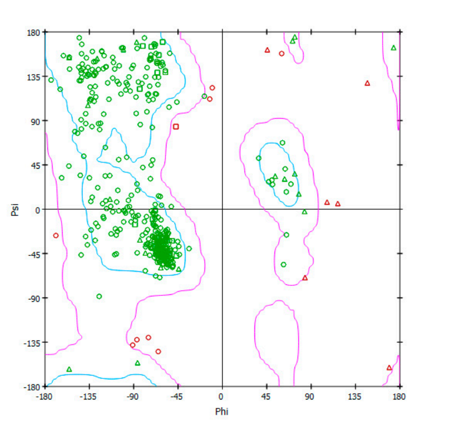
β-tubulin A0A0A0KQW7 m7	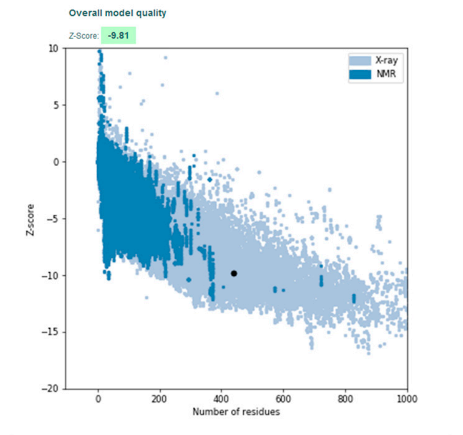	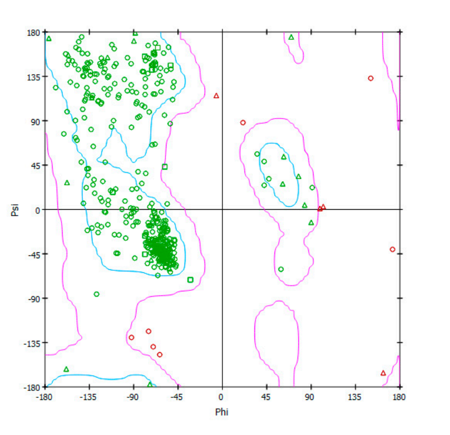

Table S4. Interaction diagrams between *C. sativus* tubulin models and the lowest-energy conformation of each cluster. Figures on the left showed the 3D interaction network view of docked COL into the CBS; hydrogen bonds and electrostatic and hydrophobic interactions can be observed. The diagrams on the right can be regarded as a 2D interaction network view between docked COL and the interacting residues of the αβ-tubulin model of *C. sativus*.

**Mode 1**	**3D binding modes**	**2D binding modes**
4O2B	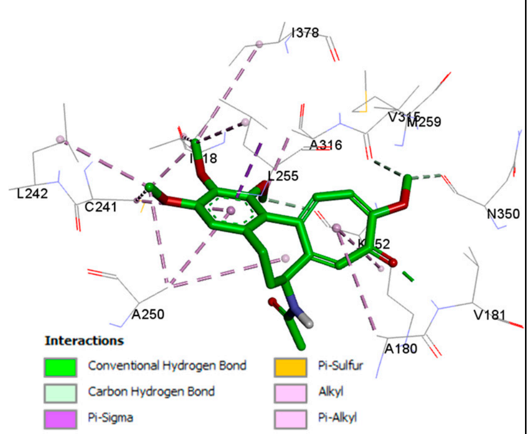	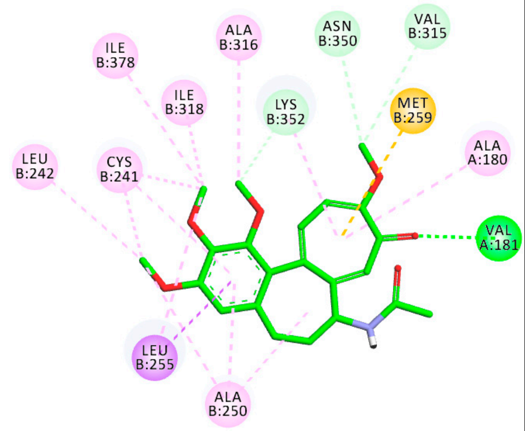

m1

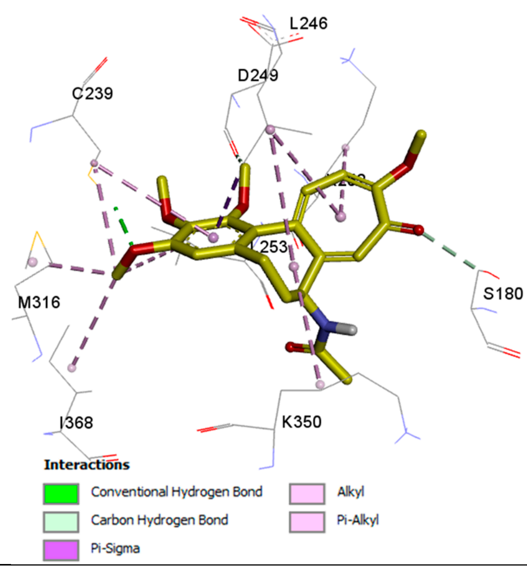
L246
D249
C239
M316
S180
K350
I368
253
Interactions
Conventional Hydrogen Bond
Carbon Hydrogen Bond
Pi-Sigma
Alkyl
Pi-Alkyl

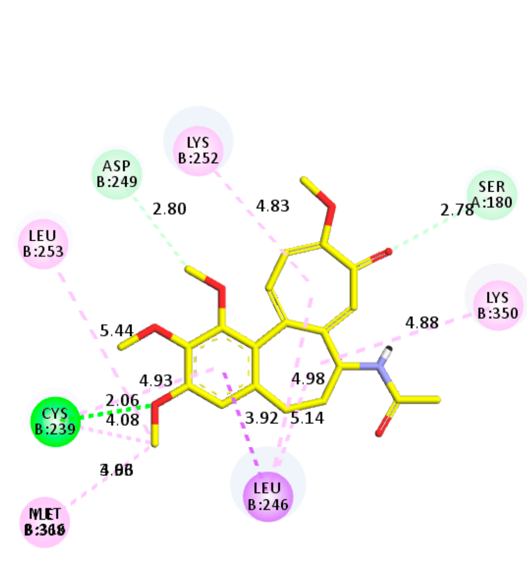
ASP B:249
LYS B:252
SER A:180
LEU B:253
LYS B:350
CYS B:239
LEU B:246
2.80
4.83
2.78
5.44
4.88
4.93
4.98
2.06
4.08
3.92
5.14

m2

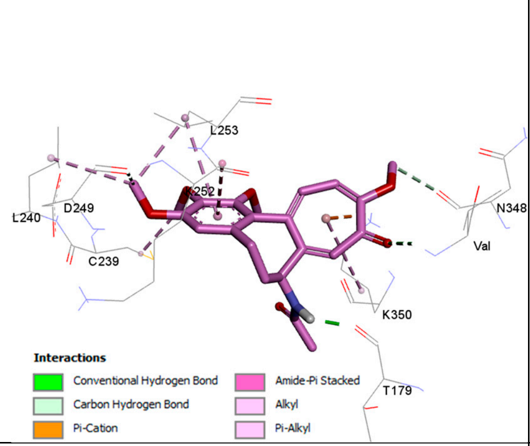
L253
L240
D249
C239
N348
Val
K350
T179
Interactions
Conventional Hydrogen Bond
Carbon Hydrogen Bond
Pi-Cation
Amide-Pi Stacked
Alkyl
Pi-Alkyl

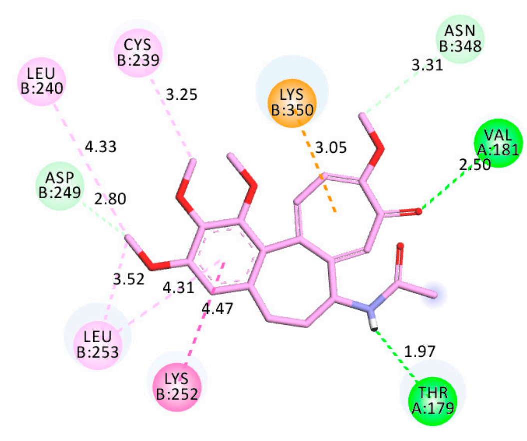
LEU B:240
CYS B:239
ASN B:348
LYS B:350
VAL A:181
ASP B:249
LEU B:253
LYS B:252
THR A:179
3.25
3.31
4.33
3.05
2.50
2.80
3.52
4.31
4.47
1.97

m3

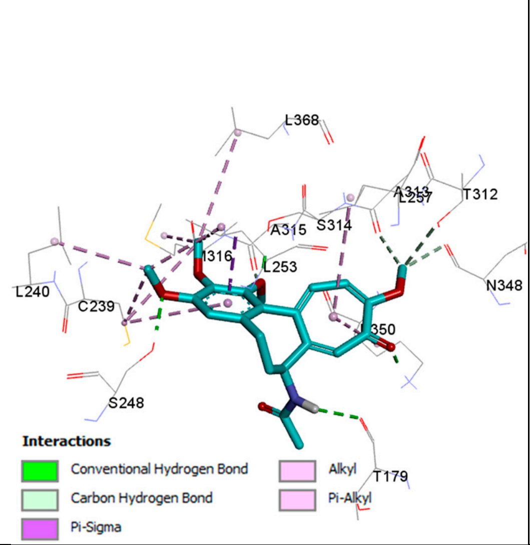
L368
A315
S314
T312
I316
L253
L240
C239
N348
S248
T179
Interactions
Conventional Hydrogen Bond
Carbon Hydrogen Bond
Pi-Sigma
Alkyl
Pi-Alkyl

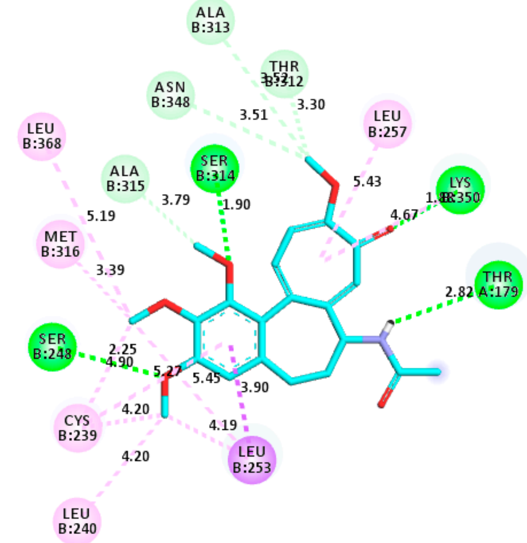
ALA B:313
THR B:312
ASN B:348
LEU B:368
LEU B:257
ALA B:315
SER B:314
LYS B:350
MET B:316
THR A:179
SER B:248
CYS B:239
LEU B:253
LEU B:240
3.51
3.30
5.43
3.79
1.90
5.19
4.67
3.39
2.82
2.25
4.90
5.27
5.45
3.90
4.20
4.19

m4

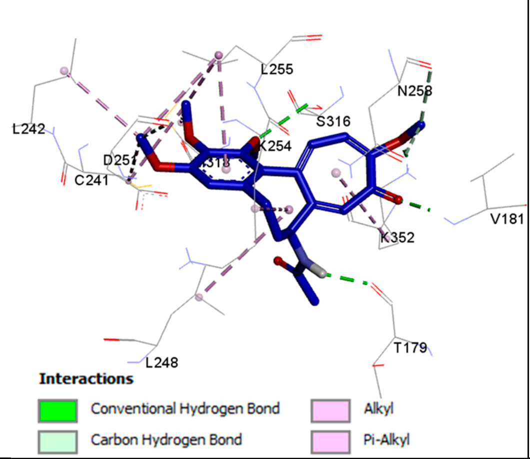

L255
N258
S316
L242
D251
K254
C241
V181
K352
T179
L248
Interactions
Conventional Hydrogen Bond
Carbon Hydrogen Bond
Alkyl
Pi-Alkyl

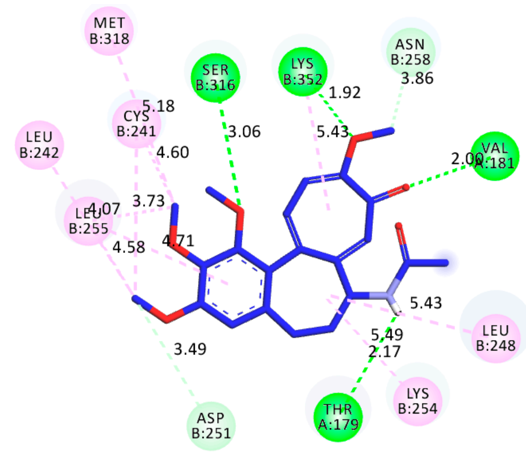

MET B:318
SER B:316
LYS B:352
ASN B:258
CYS B:241
LEU B:242
VAL A:181
LEU B:255
LEU B:248
LYS B:254
THR A:179
ASP B:251

m5

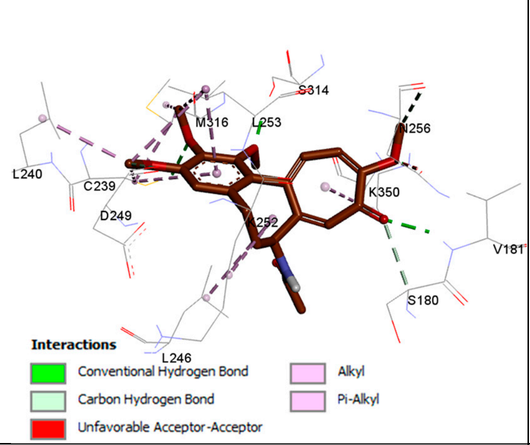

S314
M316
L253
N256
L240
C239
K350
D249
V181
S180
L246
Interactions
Conventional Hydrogen Bond
Carbon Hydrogen Bond
Unfavorable Acceptor-Acceptor
Alkyl
Pi-Alkyl

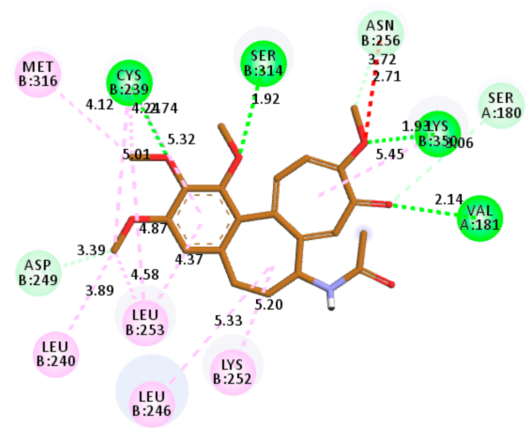

ASN B:256
MET B:316
CYS B:239
SER B:314
SER A:180
VAL A:181
ASP B:249
LEU B:253
LEU B:240
LYS B:252
LEU B:246

m6

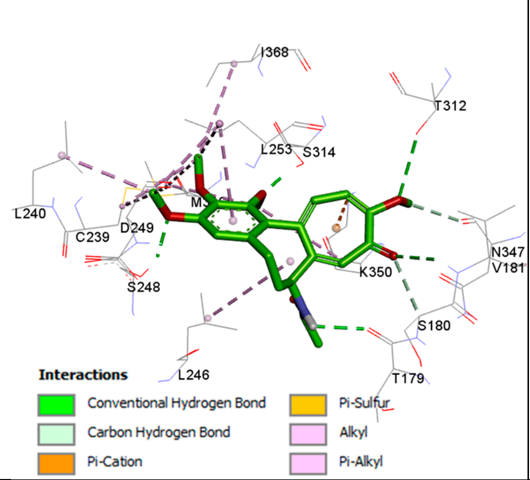

I368
T312
L253
S314
L240
C239
D249
N347
V181
K350
S248
S180
L246
T179
Interactions
Conventional Hydrogen Bond
Carbon Hydrogen Bond
Pi-Cation
Pi-Sulfur
Alkyl
Pi-Alkyl

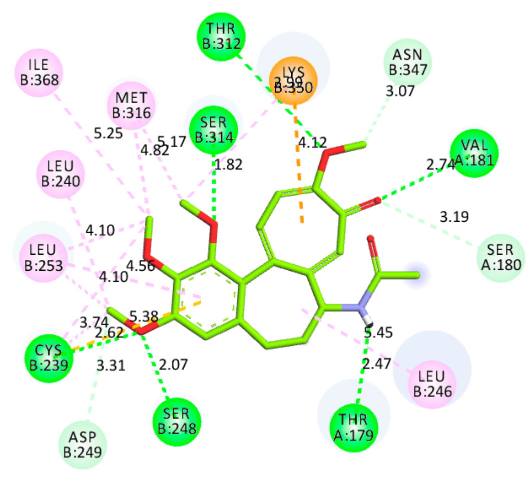

THR B:312
ILE B:368
ASN B:347
MET B:316
SER B:314
VAL A:181
LEU B:240
LEU B:253
SER A:180
CYS B:239
SER B:248
THR A:179
LEU B:246
ASP B:249

m7

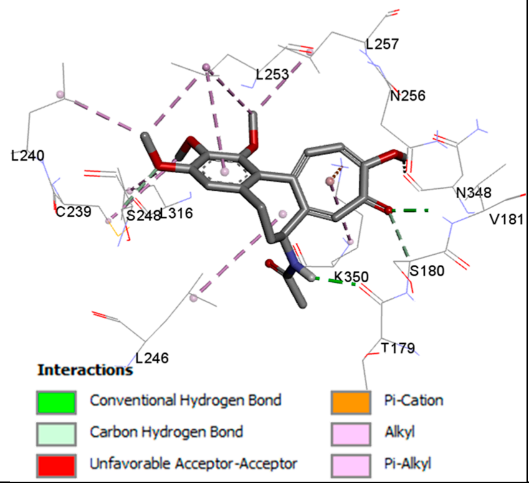
L257
L253
N256
L240
C239
S248
L316
N348
V181
K350
S180
T179
L246
Interactions
Conventional Hydrogen Bond
Carbon Hydrogen Bond
Unfavorable Acceptor-Acceptor
Pi-Cation
Alkyl
Pi-Alkyl

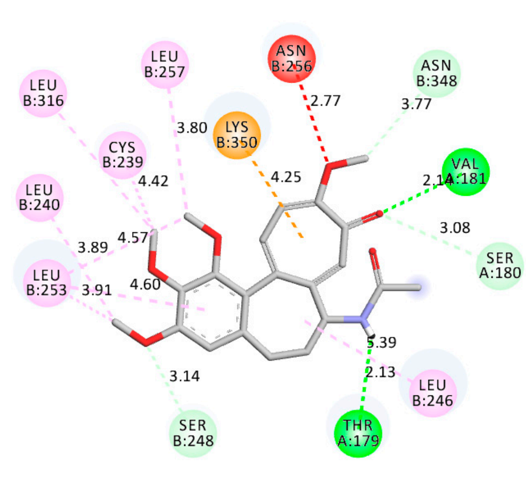
LEU B:316
LEU B:257
ASN B:256
ASN B:348
CYS B:239
LYS B:350
VAL A:181
LEU B:240
SER A:180
LEU B:253
LEU B:246
SER B:248
THR A:179
3.80
2.77
3.77
4.42
4.25
2.14
3.08
3.89
4.57
3.91
4.60
5.39
2.13
3.14
